# Proximal Protein Interaction Landscape of RAS Paralogs

**DOI:** 10.3390/cancers12113326

**Published:** 2020-11-11

**Authors:** Benoît Béganton, Etienne Coyaud, Estelle M. N. Laurent, Alain Mangé, Julien Jacquemetton, Muriel Le Romancer, Brian Raught, Jérôme Solassol

**Affiliations:** 1 CHU Montpellier, Department of Pathology and Onco-Biology, Univ Montpellier, 34295 Montpellier, France; j-solassol@chu-montpellier.fr; 2 IRCM, INSERM, Univ Montpellier, ICM, 34298 Montpellier, France; alain.mange@umontpellier.fr; 3 Department of Medical Biophysics, Princess Margaret Cancer Centre, University of Toronto, Toronto, ON M5G 1L7, Canada; Etienne.coyaud@inserm.fr (E.C.); Estelle.laurent@univ-lille.fr (E.M.N.L.); Brian.raught@uhnresearch.ca (B.R.); 4 Centre de Recherche en Cancérologie de Lyon (CRCL), INSERM U1052, CNRS UMR5286, Université Lyon 1, 69008 Lyon, France; julien.jacquemetton@lyon.unicancer.fr (J.J.); muriel.leromancer-cherifi@lyon.unicancer.fr (M.L.R.)

**Keywords:** RAS paralogs, interactome, protein–protein interaction, BioID

## Abstract

**Simple Summary:**

RAS paralogs (HRAS, NRAS and KRAS) are of major interest in biology because they are involved in developmental disorders (e.g., Costello and Noonan syndromes) and in a broad variety of human neoplasia. Many research groups have devoted tremendous efforts to deepen our understanding of the RAS proteins functions and regulations, notably through identifying their functional protein partners. However, while most of these studies were focused on pathogenic RAS mutants, much less research has been dedicated to deciphering the normal activities of RAS paralogs. However, such characterization appears as a prerequisite to clearly identify pathogenic features. We delineated and compared the wild type RAS paralogs proximal interactomes. We detected more than 800 RAS high confident proximal interactors, either shared between paralogs or unique, and validated a subset of data through proximity ligation assays-based validation. Our results describe differential interactors enrichment between RAS paralogs and uncover novel ties between RAS signaling and cellular metabolism. We believe that our findings will support further studies aiming at better understanding how RAS paralogs could be differentially involved in discrete cellular processes and could serve as a basis to template oncogenic mechanism investigations.

**Abstract:**

RAS proteins (KRAS, NRAS and HRAS) are frequently activated in different cancer types (e.g., non-small cell lung cancer, colorectal cancer, melanoma and bladder cancer). For many years, their activities were considered redundant due to their high degree of sequence homology (80% identity) and their shared upstream and downstream protein partners. However, the high conservation of the Hyper-Variable-Region across mammalian species, the preferential activation of different RAS proteins in specific tumor types and the specific post-translational modifications and plasma membrane-localization of each paralog suggest they could ensure discrete functions. To gain insights into RAS proteins specificities, we explored their proximal protein–protein interaction landscapes using the proximity-dependent biotin identification technology (BioID) in Flp-In T-REx 293 cell lines stably transfected and inducibly expressing wild type KRAS4B, NRAS or HRAS. We identified more than 800 high-confidence proximal interactors, allowing us to propose an unprecedented comparative analysis of wild type RAS paralogs protein networks. These data bring novel information on poorly characterized RAS functions, e.g., its putative involvement in metabolic pathways, and on shared as well as paralog-specific protein networks that could partially explain the complexity of RAS functions. These networks of protein interactions open numerous avenues to better understand RAS paralogs biological activities.

## 1. Introduction

The *RAS* genes were characterized in the 1980s and encode four highly related and nearly ubiquitously expressed proteins: HRAS, NRAS, KRAS4A and KRAS4B. These small GTPases are at the crossroad of a broad range of cellular signaling networks and are essential for cell fate determination through their roles in cell proliferation, differentiation and survival [1,2,3]. RAS proteins switch between the active GTP-bound and the inactive GDP-bound states, regulated by the direct interaction with Guanine nucleotide Exchange Factors (GEFs) and GTPase-Activating Proteins (GAPs) [4]. RAS regulation is dependent of multiple signals integration, and this GTP–GDP cycling enables the transduction of extracellular stimuli to intracellular signaling cascades, required for cell adaptation to environmental changes [5,6]. A fine understanding of RAS functions is particularly important given their extremely frequent constitutive activation in human cancers (e.g., non-small cell lung cancer, melanoma and bladder cancer) and developmental disorders (i.e., Costello and Noonan syndromes). KRAS mutations are considered to be responsible for approximately one million deaths per year worldwide [7,8]. Hence, RAS proteins have been extensively studied and considerable efforts have been devoted to decipher their functions and identify specific alterations. However, little progress has been made on RAS targeting, and there is still no clinical trial [9,10,11].

For many years, the high degree of sequence homology (~80% identity) of the regulatory and effector binding regions led to consider the activities of RAS paralogs as redundant [12,13]. Indeed, RAS interactors (e.g., NF1, RASAL2, RAF proteins and PI3KCA) are part of the well-described common set of upstream and downstream RAS paralogs interacting partners [6,14,15,16]. However, accumulating evidences strongly suggest distinct biological properties among HRAS, NRAS, KRAS4A and KRAS4B. First, the overall high sequence homology is misleading: the Hyper-Variable-Region, which consists of 19–20 amino acid residues, displays only 15% identity across the three paralogs and is critical for mediating post-translational modifications (PTM) and plasma membrane localization [17,18], suggesting distinct and preserved functions [19,20,21]. Second, in RAS-activated tumors, only one paralog is preferentially mutated, although they are all expressed in the matched health tissue [2,22]. Last, only KRAS4B is essential for mouse embryogenesis, but not KRAS4A, HRAS and NRAS [23,24]. Altogether, these findings prove that HRAS, NRAS, KRAS4A and KRAS4B are not fully redundant and support the concept of paralog-specific RAS functions. 

In living cells, proteins are embedded in finely tuned interaction networks at specific locations to wield their functions. Following extracellular stimuli or intracellular perturbations, the protein interaction networks are dynamically rearranged, over both time and space, to recover cell homeostasis. Each protein biological functions are intimately related to their protein partners [25]. Over the last years, RAS research has been mainly focused on their pathological roles, whereas little is known about their physiological activities and specificities, although this appears as a prerequisite to clearly discriminate between pathogenic and non-pathogenic behaviors [8,14]. Surprisingly, high-throughput in vivo proteomic analyses of RAS Protein–Protein Interactions (PPIs) are lagging behind, while technological tools to identify in vivo PPIs are rapidly evolving [26,27,28,29]. However, the characterization of RAS protein proximal landscape of interactions is an essential step towards understanding their biological functions. Proximity-dependent biotinylation identification (BioID) has emerged as a powerful tool to gain new information on protein functions, especially for proteins located in poorly soluble compartments, such as membranes [30]. Indeed, when classical techniques in which co-immunoprecipitation is followed by Mass Spectrometry (MS) are implemented to explore membrane protein interactions, PPIs are either lost during cell lysis or discarded in the non-solubilized fraction [26]. BioID is of particular interest for RAS proteins because they are anchored to the plasma membrane, and, upon activation, they trigger the translocation of proteins to the plasma membrane [31]. Moreover, other available PPI identification approaches (e.g., yeast-two hybrid system and in vitro binding assays) are not performed in living human cells and require extensive cross validation. BioID circumvents most of these caveats and enables the identification of transient, weak and poorly soluble PPIs in living cells [27]. Such PPIs are characterized by specific dissociation constants. Of the utmost interest, transient and weak interactions are pivotal to control cellular metabolism, signaling and regulation [32]. BioID allows identification of proximal partners of a protein of interest by covalently biotinylating the surrounding polypeptides within a radius of ~10 nm [33]. Thus, the detected proteins may interact physically with the prey protein (direct interaction), within a protein complex (indirect interaction) or be in the bait protein immediate proximity (no physical contact). The term interaction, when applied to BioID analysis, encompasses this variety of protein–protein relationships. BioID has been successfully employed to study cilia architecture and specific signaling cascades of oncogenic fusion proteins. These findings support this technology efficiency to uncover novel PPIs and protein functions [34,35,36].

In this study, we used BioID to determine the PPIs of wild type HRAS, NRAS and KRAS4B, the major KRAS transcript, to propose a comprehensive picture of RAS paralogs proximal interactions landscape in a non-cancerous human cellular context. 

## 2. Results

### 2.1. The RAS BioID-Based Interactome

**Figure 1:**
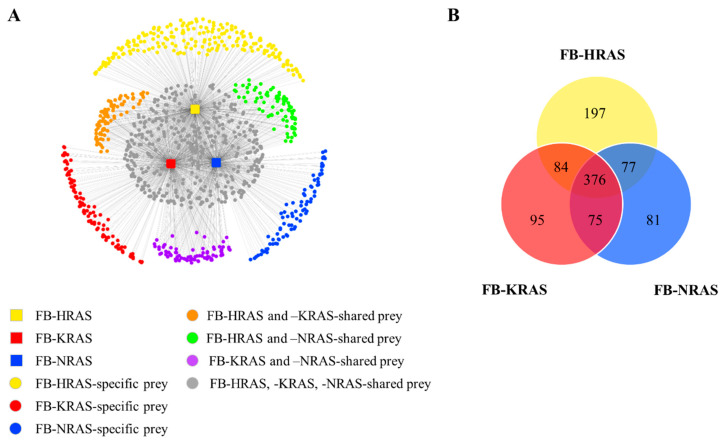
The RAS interactome by BioID. (**A**) The network of RAS protein–protein interactions includes shared and specific interactors (prey) for the three indicated RAS paralogs. The prey protein location is based on their total spectral count, using the edge-weighted spring-embedded layout algorithm (Cytoscape v3.2.1). (**B**) Venn diagram showing the number of identified shared and specific interactors for the three indicated RAS paralogs. FB, FlagBirA* dual tag.

To gain information on RAS PPIs landscape, a BioID analysis was performed in Flp-In T-REx 293 cells stably transfected by the three RAS paralogs (HRAS, NRAS and KRAS4B). In total, 985 high confidence RAS-proximal interactors were identified in this analysis (Table S1). This represents more than four times the total number of RAS interactors referenced in the BIND, BioGRID and HPRD databases. This large number of interacting partners included shared as well as specific RAS paralog interactors (Figure 1). We identified 734, 609 and 630 proteins interacting with HRAS, NRAS and KRAS4B, respectively (SAINT score ≥ 0.75). Although most of the identified interactors (*n* = 376) were found in the proximity of all three paralogs, each paralog displayed a set of unique interactors. HRAS identified the largest network of unique PPIs (*n* = 197 partners), compared to KRAS4B (*n* = 95) and NRAS (*n* = 81) (Figure 1). The list of interactors included previously reported RAS partners, e.g., BRAF, CRAF, PI3KCA, NF1 and RASAL2 [37,38]. The comparison between our results and a previous BioID analysis of wild type and mutated RAS proteins in cancer cells [39] showed a remarkable interactomes overlap (more than 50%), including the mTORC2 complex, supporting the robustness of this approach. However, our dataset was characterized by a greater overlap with known RAS interactors reported in databases (*n* = 30 versus 17 proteins) (Figure S1). 

**Figure 2:**
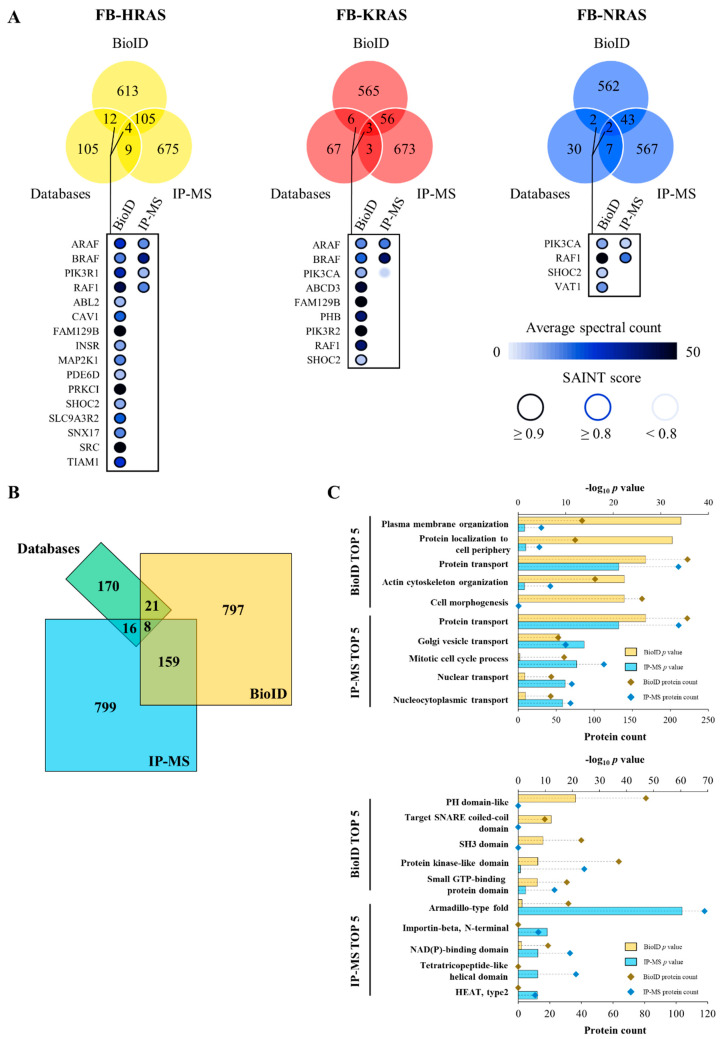
Comparison of the RAS networks identified with BioID and IP-MS. (**A**) Venn diagrams of the shared and specific interactors identified by IP-MS and BioID, as well as the overlap with RAS interactors referenced in the BioGRID, MINT and HPRD databases. The average spectral counts and SAINT scores of selected interactors are described using the indicated color codes. (**B**) Venn diagram of the putative RAS interactors identified by BioID and IP-MS, as well as those referenced in the BIND, BioGRID and HPRD databases. (**C**) (Top) Comparison of the top five Gene Ontology Biological Process terms of the putative RAS interactors identified by BioID and IP-MS; and (Bottom) comparison of the top five protein domains in the putative RAS interactors identified by BioID and IP-MS. FB, FlagBirA* dual tag.

To compare BioID results with those obtained using the conventional immunoprecipitation (IP) approach, RAS interactors were identified in the same cells by anti-Flag IP followed by MS (IP-MS) after tetracycline induction and biotin-labeling, as before. IP-MS led to the identification of 982 high-confidence RAS interactors (Table S2). As expected, the proteins identified by BioID only partially overlapped with those identified by IP-MS, regardless of the RAS paralog considered. Specifically, only approximately 15% of the HRAS interactors detected by BioID were also detected by IP-MS, and this percentage was even lower for KRAS4B and NRAS (9% and 7%, respectively) (Figure 2A). However, both BioID and IP-MS identified RAF kinases and PIK3 proteins as high-confidence interactors in each paralog dataset. These protein families are members of the mitogen-activated protein kinase (MAPK) and PIK3/Akt signaling pathways, which both belong to major signaling cascades triggered by RAS activation. MAPK and PIK3 members are also reported as RAS interactors in the BioGRID, BIND and HPRD databases.

### 2.2. The BioID and IP-MS Analyses Identified Different Subsets of RAS Interactors

The low overlap of the BioID and IP-MS datasets suggested the existence of two groups of RAS interactors, likely dependent on the biochemical features of these approaches. Indeed, only 167 proteins were present in both datasets, whereas 818 and 815 interactors were specific to the BioID and IP-MS datasets, respectively (Figure 2B). Comparison of the Gene Ontology (GO) Biological Process (BP) analysis results for the BioID and IP-MS RAS interactors (top five ontology terms) clearly identified distinct enrichments. In line with RAS localization, the BioID identified interactors were mostly related to the plasma membrane or involved in membrane architecture regulation (Figure 2C, top). Indeed, all top five GO BP terms were linked to plasma membrane organization, protein transport or actin remodeling. With the exception of “Cell morphogenesis”, all these BP terms were also present in the IP-MS dataset, but with much lower significance. This is particularly striking for the “Plasma membrane organization” term that showed a *p* value of 5.6 × 10^−35^ in the BioID and of 4.4 × 10^−2^ in the IP-MS dataset. This result was expected because of BioID suitability to identify membrane protein interactions. Conversely, IP-MS was more effective at identifying proteins involved in transport, interaction with the Golgi apparatus, an organelle required for RAS PTMs, nuclear trafficking and mitotic cell cycle process. Of note, this last term was not significant in the BioID dataset GO analysis. Considering RAS localization at the plasma membrane, the high significance of nuclear trafficking and mitotic cell cycle process categories is surprising; however, RAS proteins trigger a variety of signals that end inside the nucleus, thus possibly explaining this result. Overall, the top five GO BP terms identified using the IP-MS dataset were less relevant in view of the known RAS functions and localization. Additionally, to explain the low overlap of the detected proteins using these two approaches, we selected the non-overlapping proteins to investigate the corresponding enrichments, with a specific focus on cellular localization and biological processes. As expected, this analysis showed a significant enrichment of proteins localized to the plasma membrane and cell junction for BioID when compared to IP-MS. To investigate this discrepancy for protein localized in other compartments, we selected the cytosolic proteins identified and analyzed the related enrichments in biological processes for both techniques. Among the top ten GO BP, six terms encompassing 159 unique proteins out of the 241 proteins identified were assigned to signal transduction and regulation of GTPase activity for the proteins detected with BioID, while none of these terms were identified for the proteins detected with IP-MS. Such regulations are mainly characterized by transient and weak interactions, which are more difficult to capture using IP-MS (Tables S3 and S4).

Protein domains are at the core of PPIs and allow proteins to selectively interact and ensure cellular functions. As RAS activation and signaling are mediated by proteins harboring kinase, SH2, SH3 or PH domains, the prevalence of these domains in the BioID and IP-MS datasets was investigated using the InterPro database. This analysis showed that, in the BioID dataset, interactors with domains for plasma membrane targeting (PH domain-like), membrane trafficking domains (SNARE coiled-coil domain) and cell signaling related domains (SH3-domain, kinase-like domain and small GTP-binding protein domain) were the most common (Figure 2C, bottom). Altogether, the identified protein domains gave a more comprehensive picture of RAS mechanisms of activation and signaling (e.g., domains involved in membrane interaction, signal transduction and vesicle trafficking) and were also in line with the known RAS properties. Interestingly, these domains were poorly represented in the IP-MS dataset. Specifically, the PH domain-like, SNARE coiled-coil domain and SH3 domain were not identified, whereas enrichment for the protein-kinase-like domain was not significant, although it is one of the major domains that triggers RAS activity. Overall, with the exception of the small GTP-binding protein domain, the IP-MS dataset described a different aspect of RAS interactions. Indeed, the enriched domains were related to nuclear trafficking (N-terminal domain of importin-beta), cell cycle regulation and transcriptional control (tetratricopeptide-like helical domain) and intracellular transport (HEAT type 2 domain). The Armadillo-type fold domain, the most enriched in the IP-MS dataset, is present in signaling proteins (e.g., PIK3 proteins, small Rho GTPases and members of the Ras superfamily) and is involved in cytoskeleton remodeling [40].

### 2.3. The BioID RAS Interactors Are Implicated in Known RAS Pathways

RAS proteins are considered to be at the core of a signaling platform involving different protein families (e.g., Receptor Tyrosine Kinases (RTKs), GAPs, GEFs) and different signaling pathways (i.e., MAPK, PIK3/Akt, RAP1, calcium signaling, endocytosis and actin cytoskeleton regulation). Using the KEGG database, the RAS proximal interactors identified were mapped to the known RAS pathways (Figure S2). This map showed that the identified interactors were implicated in all known RAS activities and included regulatory proteins and effector pathways. Specifically, the BioID dataset included 17 RTKs, 3 GEFs and 4 GAPs (i.e., 25%, 11% and 19% of all members of these three families, respectively). Although RTKs generally require adaptor proteins and therefore may not physically interact with RAS proteins to promote their functions, they are essential for RAS activity and remain in their close proximity. Indeed, EGFR was identified by BioID, but is not referenced as a direct RAS interactor. Other EGFR family members were identified, such as ERBB2 and ERBB4. We also identified proximal GAPs and GEFs, such as GAB1 and NF1, respectively. RAS proteins signal through MAPK, PIK3/Akt, RAP1 and calcium pathways and are involved in actin cytoskeleton and endocytosis regulation. As expected, the BioID dataset included proteins related to these pathways (between 6% and 26% of all proteins referenced for these pathways, and notably 6% and 13% of the MAPK and PIK3/Akt pathway members, respectively). ERK proteins (i.e., MAPK1 and MAPK3) are not considered to be in direct proximity of RAS and, accordingly, were not present in the BioID dataset. The identified PIK3/Akt signaling proteins included MAPK signaling factors (i.e., RAF1, MAP2K1 and MAP2K2) and also specific PIK3 family members, such as PI3K catalytic subunit alpha and beta (PIK3CA and PIK3CB) and regulatory subunits 1, 2 and 3 (PIK3R1, PIK3R2 and PIK3R3). PIK3 is the first component of the PIK3/Akt cascade and is considered as a direct RAS interactor. Interestingly, MTOR was identified in close proximity of all RAS paralogs. Moreover, the BioID dataset included five of the seven members of the 14-3-3 family (YWHA proteins) that have not been reported as RAS interactors to date. Finally, actin cytoskeleton and endocytosis regulation were the two most represented processes, with 26% and 22% of the identified RAS interactors assigned to these categories, respectively (Figure S2 and Table S5). Actin cytoskeleton regulation is governed through multiple crosstalks between signaling and cytoskeleton-related proteins, many of which were identified as RAS interactors: RAF proteins, 11 integrins, PI3K regulating and catalytic subunits and cytoskeleton proteins (e.g., actin and radixin). Endocytosis-related factors were RAB, SNX, CHMP and VPS protein families and components of the ubiquitin system. Overall, the RAS proximal protein set thus appears relevant in view of the RAS known signaling and regulation.

### 2.4. RAS Interactors Wire Most of the Cellular Compartments

**Figure 3:**
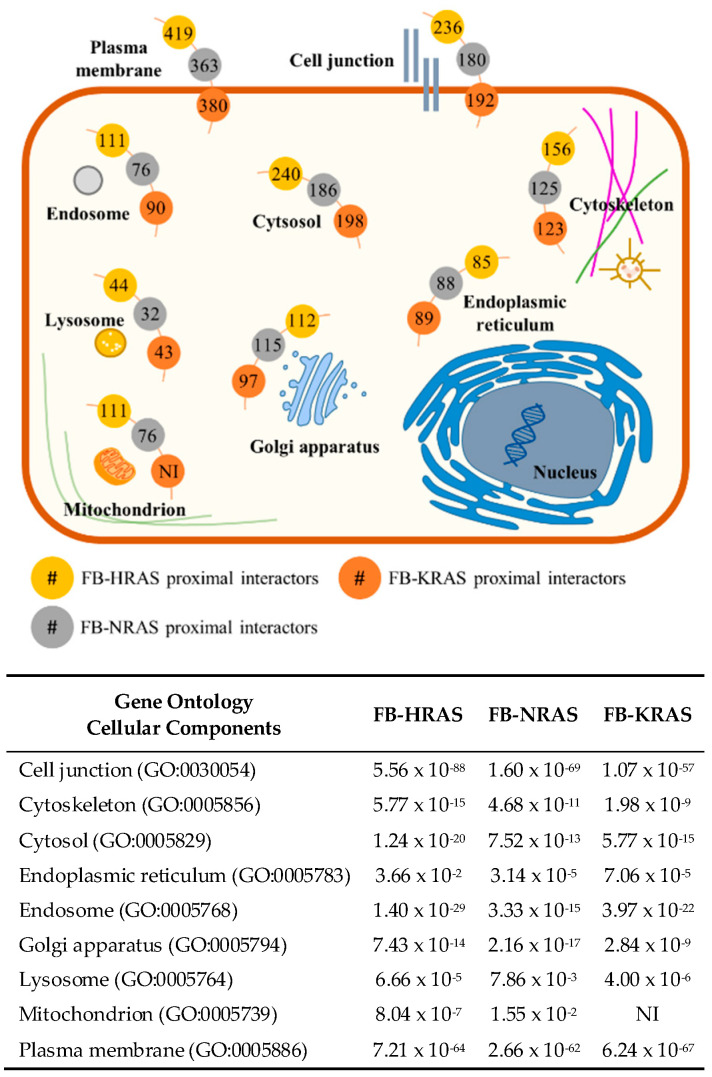
Cellular localization of the RAS proximal interactors identified by BioID: (left) cell mapping of each RAS interactor determined by Gene Ontology analysis focused on cellular components; and (right) associated *p* values calculated for each cellular components and RAS paralog. FB, FlagBirA* dual tag; NI, not identified.

Then, a GO analysis focused on cellular components (CC) was performed to map the localization of the RAS proximal interactors identified by BioID. For all three RAS paralogs, interactors were mostly mapped at plasma membrane, cell junctions, cytoskeleton and cytoplasm, with the exception of mitochondrion for KRAS4B (Figure 3). However, the more specific ontology term “mitochondrial outer membrane” was significant and included 14 KRAS4B interactors. Overall, this analysis showed the strong connection with proteins that are localized at the plasma membrane or involved in cell junctions (*p* value < 10^−55^). Lysosome was less represented, but still significant for all RAS paralogs. These findings might be explained by the fact that biotin activation requires ATP, and lysosomes are specifically rich in ATPases to efficiently degrade proteins and characterized by an acidic pH that may alter BirA* enzymatic activity. In summary, this analysis showed that RAS partners target most cellular compartments, creating a dense and dynamic network of interactions. This complexity is consistent with the specific place of RAS proteins at the crossroad of many pathways and emphasizes the complexity of RAS functions.

### 2.5. RAS Paralogs are Strongly Connected with Metabolism

**Figure 4:**
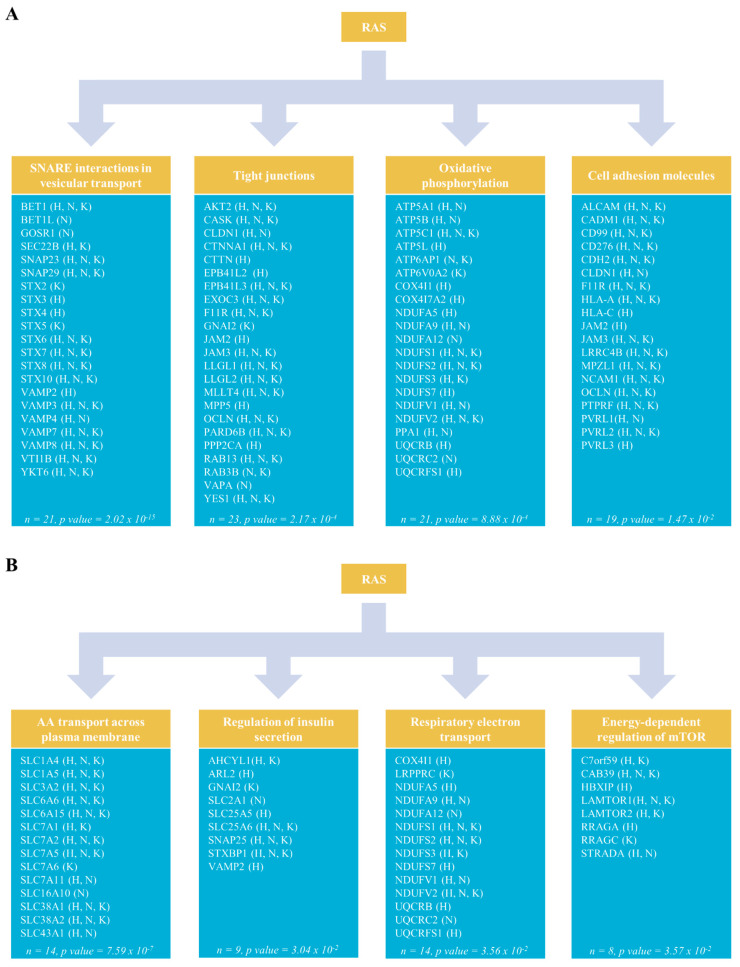
Specific pathways of the RAS proximal interactors: (**A**) The KEGG pathway analysis identified SNARE interaction in vesicular transport (hsa04130), tight junctions (hsa04530), oxidative phosphorylation (hsa00190) and cell adhesion molecules (hsa04514) as significant RAS proximal interactor pathways; and (**B**) the Reactome pathway analysis identified the close proximity of RAS paralogs with proteins involved in the following metabolism-related pathways: amino-acid (AA) transport across the plasma membrane (HSA-352230), regulation of insulin secretion (HSA-422356), respiratory electron transport (HSA-611105) and energy-dependent regulation of mTOR by LKB1-AMPK (HSA-380972). H, FB-HRAS proximal interactor; N, FB-NRAS proximal interactor; K, FB-KRAS proximal interactor; FB, FlagBirA* dual tag.

In addition to well-known RAS pathways, four other significant pathways were identified: SNARE interactions, tight junctions, cell adhesion molecules and oxidative phosphorylation (Figure 4A). The identification of pathways related to cell junctions and membrane trafficking was expected due to RAS protein localization and the PTMs occurring during their maturation [41,42]. However, the identification of 21 RAS interactors implicated in oxidative phosphorylation was surprising. In this latter pathway, the main RAS interactors were proteins from the ATP synthase complex (*n* = 5) and from the NADH-ubiquinone oxidoreductase complex (*n* = 9). This finding was confirmed by analysis of the BioID dataset using Reactome (Figure 4B) that identified the respiratory electron transport pathway, which corresponds to the KEGG oxidative phosphorylation pathway. Three other Reactome energy-related pathways were also enriched, thus highlighting a strong connection between RAS and metabolism. This last analysis thus identified the “Amino acid transport across the plasma membrane”, “Regulation of insulin secretion” and “Energy dependent regulation of mTOR by LKB1-AMPK” categories. Together with the “Respiratory electron transport” ontology enrichment, it suggests an intimate interplay between RAS cellular signaling and metabolism. This interplay encompasses vesicle trafficking, membrane transport, MTOR cell signaling and mitochondrial activity. Moreover, it was recently shown that SNARE proteins are involved in insulin vesicle secretion [43], and thus could coordinate this interconnection. Additionally, enzymes involved in the central carbon metabolism were also detected, such as glycolytic enzymes (e.g., PFKP, ENO1 and GAPDH) and PGD, a proximal interactor specific to KRAS4B which is involved in the pentose phosphate pathway. Overall, the metabolic pathways detected involved proximal interactors specific to one or more RAS paralog, suggesting a coordinated and complementary activity of the RAS proteins with metabolic regulations. None of these RAS proximal partners was previously described, and the close proximity between RAS and cellular energy processes opens new hypothesis on RAS cellular functions.

### 2.6. RAS Paralogs Exhibit Specific Functions

**Figure 5:**
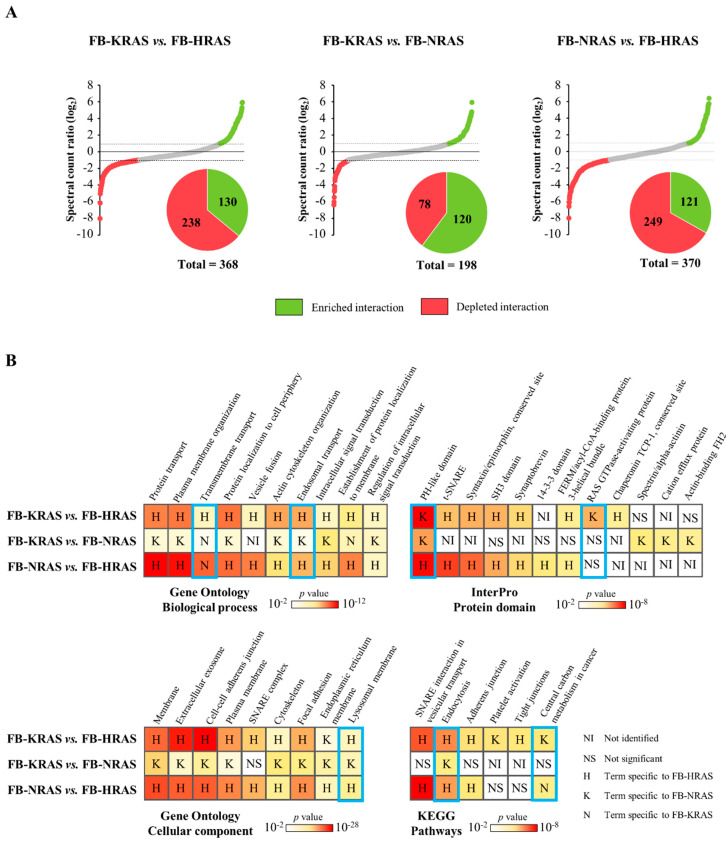
Features of RAS paralog-proximal interactors. (**A**) Based on the spectral count ratio of each interactor, a protein was considered as specific to one RAS paralog when at least two-fold more prey peptides were measured for that bait, and statistically significant (*p* value < 0.05) as determined by student’s *t*-test. (**B**) Comparison of Gene Ontology (GO) Biological Process and Cellular Component, InterPro protein domain, and KEGG pathway term enrichment for the interactors specific to each RAS paralog. The blue rectangles highlight terms that depict the most important differences between the compared RAS paralogs. FB, FlagBirA* dual tag.

The semi-quantitative BioID analysis, based on the spectral counts for each PPI, allowed determining the RAS paralog specificities (Figure 5A and Table S6).

Overall, compared with HRAS, proximal interactors linked to cellular membranes and actin cytoskeleton were less common in the KRAS4B and NRAS samples (Figure 5B). Indeed, interactors annotated to GO BP terms referring to membranes (e.g., plasma membrane organization, protein localization to cell periphery, vesicle fusion and actin cytoskeleton organization) and to the relevant GO CC terms, InterPro protein domains and KEGG pathways (e.g., plasma membrane, cell–cell adherent junction, cytoskeleton and the protein domains t-SNARE, syntaxin/epimorphin and chaperonin TCP-1) were more enriched with HRAS than with KRAS4B or NRAS. However, the proteins DAB2IP, IQGAP1, IQGAP2, NF1 and PLXNB2, which harbor a GAP protein domain, were identified mainly with KRAS4B rather than HRAS. Interestingly, the GAP ontology term was not significant when HRAS PPIs were compared to NRAS PPIs, suggesting a closer connection of KRAS4B to these RAS regulating proteins. The PH-like domain, harbored by membrane-interacting proteins and several RAS interacting partners (e.g., GAB1 and TRIO), was also significantly enriched, and more proteins with this domain were enriched with KRAS4B than with HRAS. Compared with HRAS, both KRAS4B and NRAS were enriched for the “Central carbon metabolism in cancer” pathway, which includes proteins from the PIK3/Akt and MAPK pathways, glucose transporters, metabolic enzymes and tyrosine kinase receptors, all being frequently activated in cancer. Both KRAS4B and NRAS interactomes were enriched for these factors, particularly for proteins from the PIK3/Akt cascade (e.g., AKT1, PIK3CA and MTOR). These observations support a possible link between RAS and metabolism (Figure 4) and emphasize the specific involvement of NRAS and KRAS4B in this interplay. Despite the functional similarity of NRAS and KRAS4B, KRAS4B displayed a stronger link with vesicle trafficking than NRAS. Indeed, interactors annotated to the GO BP terms endosomal transport, endocytosis pathway and to the GO CC terms lysosomal membrane were enriched with KRAS4B, particularly proteins from the VPS and SNX families. Only interactors assigned to the GO BP term transmembrane transport were significantly enriched with NRAS compared to KRAS4B and HRAS. This term included membrane transporters from the protein families SLC25 (phosphate mitochondrial transport), SLC30 (zinc balance) and SLC35 (nucleotide sugar transport) [44,45,46].

Then, to describe functional specificities of each RAS paralog, the same method was used to determine the PPIs and associated functions for each paralog (Figure S3 and Table S7). The top five GO BP terms for HRAS-specific proximal interactors were related to membrane processes, such as protein transport, membrane fusions, endocytosis and exocytosis. Accordingly, the SNARE pathway (not shown) and the t-SNARE, synaptobrevin and syntaxin/epimorphin domains were significantly enriched in the HRAS proximal interactome (Table 1 and Table 2). Few significant ontology terms were identified for KRAS4B and NRAS. Nevertheless, NRAS was strongly connected with transmembrane transport through its proximal interactions with transporters from the SLC25, SLC30 and SLC35 families and glucose transporter 1 (SLC2A1) (Table 1). The CRAL-TRIO protein domain was the only significant ontology term identified for KRAS4B-specific interactors (Table 2).

Overall, HRAS seemed more related than the other paralogs to membrane and vesicle trafficking proteins, whereas NRAS was connected to membrane transporters that regulate metabolites and inorganic molecule fluxes. Finally, KRAS4B displayed strong and specific enrichment with signaling regulating proteins, emphasizing the signaling-hub role of this RAS paralog.

### 2.7. Dataset Validation

**Figure 6:**
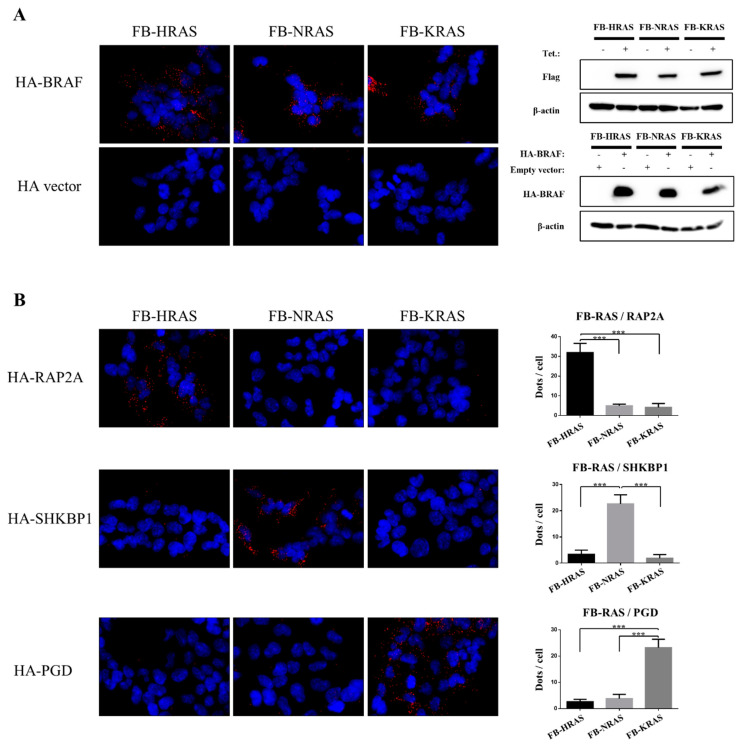
Validation by PLA of specific RAS paralogs interactors. (**A**) Detection by PLA of the interaction between the indicated FB-RAS paralogs and HA-BRAF (positive control). PLA was performed with anti-Flag and anti-HA antibodies. The detected pairs are represented by red dots. Nuclei were counterstained with DAPI (blue). Expression of HA-BRAF and the tetracycline-dependent expression of the FB-RAS paralogs were confirmed by Western blotting. The PLA negative control was performed using FB-RAS-induced cells transfected with the empty HA vector. (**B**) PLA detection of the specific FB-HRAS, FB-NRAS and FB-KRAS interaction with HA-tagged RAP2A, SHKBP1 and PGD, respectively. PLA was performed as in A and the ectopic expression of the HA-tagged interactors was validated by Western blotting (Figure S4). Quantification of the dots per cell was performed using ImageJ and the plugin “Counter cells”. Data are the mean ± SEM of 100 cells/condition. ***, *p* value ≤ 0.0001 (Student’s *t*-test). FB, FlagBirA* dual tag; Tet., tetracycline.

To validate RAS paralogs’ specific proximal interactions, proximity ligation assay (PLA) was implemented to detect PPIs within a radius of ~30 nm. Since the PLA technique allows for detection of proteins within a proximal environment, regardless of the compartment solubility, it represents an orthogonal technique of choice for BioID results validation. Besides BRAF (positive control), eight HA-tagged putative proximal interactors were selected on the basis of their spectral counts and SAINT score: three FB-HRAS, two FB-NRAS and three FB-KRAS4B interactors (Table S8) that are involved in different cellular processes, such as metabolism, cell signaling and vesicular transport. BRAF interacted with all three RAS paralogs (Figure 6A). FB-HRAS reacted with HA-RAP2A and HA-S100A11, FB-NRAS detected HA-EBP and HA-SHKBP1 and FB-KRAS4B detected HA-PGD (Figure 6B and Figure S4), confirming our BioID results. Conversely, the interactions of LASP1 with HRAS and TRIM13 and VPS35 with KRAS4B were not confirmed by PLA.

## 3. Discussion

Understanding the RAS paralogs’ interaction landscape is a major challenge in cell biology. The identification of RAS interactions has been difficult, particularly because RAS proteins show a high degree of homology and similarities of function and are orchestrating a complex cell signaling hub. Moreover, many RAS interactors are localized in poorly soluble cellular compartments, thus hindering their isolation and identification by standard IP-based techniques.

Here, we demonstrated that BioID combined with semi-quantitative MS can be employed to identify the three main RAS paralogs PPIs in non-malignant cells. Indeed, this method identified a number of reported RAS-interacting proteins, supporting our results, and new putative interactors that could link RAS to several novel biological functions. Comparison with the results of an independent BioID-based analysis of the RAS proximal proteome showed an overlap of more than 50% [39], confirming the robustness of proximal proteomic techniques. Most of the observed differences between these BioID datasets could come from the different cell types used: non-malignant Flp-In T-REx 293 cells in our study versus three different cancer cell lines in the previous BioID study. Cancer cell lines harbor many mutations and express a different range of protein species compared to HEK293. This results in altered signaling, which is likely to impair RAS proximal interactomes. Another study used the mutated RAS paralogs as bait proteins in cancerous cell lines to decipher its pathological PPIs networks [47]. These studies, taken together with our dataset, provide a broad coverage of the RAS paralogs ability to interact with diverse effectors both in cancerous and non-cancerous contexts. Using HEK293 as a non-cancerous bioreactor, we hypothesize that our data better inform on the physiological RAS activity.

An important limitation of IP-based approaches for PPIs characterization sits in the constraint of maintaining PPIS along the solubilization and purification process, precluding the detection of PPIs within poorly soluble compartments. As BioID utilizes a covalent biotin modification to mark bait protein interactors in living cells, it is no longer required to preserve PPIs upon purification and harsh lysis techniques can be employed to thoroughly solubilize polypeptides regardless of their cell subcompartment solubility. For the reasons mentioned above, the RAS interactors identified by IP-MS were not enriched for the plasma membrane organization category, unlike what we observed using BioID. Moreover, when we separately analyzed the GO BP related to the cytosolic proteins detected with BioID and IP-MS, we observed that BioID is more effective at detecting proteins involved in signal transduction and signal regulation. Indeed, 159 proteins out of the 241 cytosolic proteins were identified in such cellular processes. Considering the transient interactions of signaling proteins and their putative recruitment at cortical signaling hubs, their identification using IP-MS appeared challenging. Indeed, no term related to signal transduction and regulation were enriched among the cytosolic proteins detected using IP-MS. The discrepancy between BioID and IP-MS has already been pointed-out in several studies and has reinforced the need to complete our view on protein interactions using proximity labeling techniques [30,48,49,50]. This is particularly important for proteins involved in complex and poorly soluble cell signaling hubs like the RAS paralogs. The BioID features support the relevance of such technique to detect RAS interactions at the cell cortex and reinforce our confidence in our dataset. Moreover, well-known RAS regulators were identified (e.g., GAP and GEF proteins), as well as components of the RAS major downstream signaling pathways (e.g., MAPK and PIK3/Akt pathway). In addition, we detected RAF or MEK proteins, further stressing the efficient labeling spectrum of BioID.

Interestingly, analysis of the BioID dataset highlighted a link between the three RAS paralogs and proteins involved in mitochondrial metabolism and oxidative phosphorylation pathway. Interestingly, a relation between deregulation of mitochondrial functions and KRAS mutations and RAS direct interaction with the mitochondrial outer-membrane following PKC phosphorylation in cancers were already reported [51,52,53]. However, little is known about the physiological role of RAS at the mitochondria and in the respiratory chain. The identified proximal interaction between RAS and proteins of the ATP synthase complex and NADH-ubiquinone oxidoreductase complex could explain these observations and deserves further investigations. Indeed, while these complexes appeared to be more related to HRAS, we also detected specific proximal interactions of mitochondrial components with NRAS and KRAS4B, suggesting a complementary aspect of the RAS proteins in such signaling. Additionally, a recent study has involved KRAS4A in carbon metabolism through the direct regulation of the glycolytic enzyme HK1 [54]. This study is of great interest since it is the first proof of a direct regulation of a metabolic enzyme by a GTPase. In our study, enzymes from this specific pathway were also identified, such as the second key glycolysis regulator PFKP. Together with HK1, it controls the two first irreversible metabolite conversion and therefore both factors are critical for controlling the carbon flux through the glycolysis pathway [55]. In contrast to this study, we focused on the major transcript KRAS4B within a non-cancerous cellular background. This could explain why HK1 was not detected. However, we validated the specific proximal interaction of KRAS4B with PGD. PGD is involved in the pentose phosphate pathway, and may represent another relation between cellular signaling and a major energetic pathway. Even if we did not identify HK1 as a RAS proximal interactor, these findings support the hypothesis of a role of RAS paralogs and metabolism [56,57,58].

Among the three paralogs, KRAS is the most frequently mutated protein in cancers, followed by NRAS [59]. While all RAS paralogs are regulated by GAPs and GEFs, our proximal interactome data clearly show that KRAS4B is the most linked to proteins that harbor a GAP domain (e.g., DAB2IP, NF1, RALGAPA1, RASA2, RASA3 and RASAL2) and GEF domain (e.g., DENND3, DENND5B, KALRN and TRIO), suggesting a more complex regulation of this specific paralog. Additional studies are required to better understand the specific regulations of the three RAS paralogs, but our findings suggest a greater regulation complexity of KRAS4B versus HRAS and NRAS. Interactors harboring the CRAL-TRIO domain could also be of utmost interest. This protein domain is present in NF1, MOSPD2, KALRN and TRIO and is required for protein–lipid interactions. While MOSPD2 is poorly described, KALRN and TRIO are specific GEFs for Rho and Rac GTPase activation [60]. Through their GEF activities, and given the cortical KRAS4B localization and the lipid-binding activity of KALRN and TRIO, these two proteins could indirectly impact KRAS4B regulation [61,62]. In addition, KALRN and TRIO also harbor a SH3 and kinase domain that is involved in cellular signaling and regulation.

Altogether, our data shed light on a novel aspects of RAS paralogs biological activities in a non-cancerous context and may serve as a basis to further understand the pathological role of RAS proteins in cancer.

## 4. Materials and Methods

### 4.1. Cell Culture

The cell lines used in this study were made from the commercial cell line Flp-In™ T-REx™ (Invitrogen, Santa Clara, CA, USA). The cell lines tested negative for mycoplasma infection.

Flp-In T-REx 293 cells were bought from Invitrogen (R75007) and cultured in DMEM media supplemented with 10% fetal bovine serum (FBS) and incubated in 5% CO_2_ at 37 °C. After transfection of the pcDNA5 FRT/TO FlagBirA* expression vector, cells were grown with 0.4% Hygromycin B for selection.

### 4.2. Cloning

All PCR reactions were performed using Q5 DNA polymeRASe (NEB) according to the manufacturer’s instructions. Primers, destination plasmids, cloning sites and DNA templates are listed in Table S9. PCR products were digested with AscI and NotI (NEB) and ligated in the destination plasmid using T4 DNA ligase (NEB) following the manufacturer’s instructions.

### 4.3. BioID

The full-length wild type human HRAS (NM_005343.3), NRAS (NM_002524.4) and KRAS4B (NM_004985.4) coding sequences were PCR-amplified and cloned in the pcDNA5 FRT/TO FlagBirA* expression vector. As RAS proteins undergo several PTMs to be functional, and cleavage of a targeting motif at the C terminus followed by prenylation is required for their targeting to the plasma membrane [63], the FlagBirA* tag was fused to the N terminus of the RAS proteins. Using the Flp-In system (Invitrogen, Santa Clara, CA, USA), Flp-In T-REx 293 cells that stably express wild type FlagBirA*-HRAS, FlagBirA*-NRAS and FlagBirA*-KRAS4B were generated. After selection (DMEM + 10% FBS + 200 µg/mL hygromycin B), 10 × 150 cm² plates of subconfluent (60%) cells were cultured in complete medium supplemented with 1 µg/mL tetracycline (Sigma) and 50 µM biotin (BioShop, Burlington, ON, Canada) for 24 h. Cells were collected and pelleted (2000 rpm, 3 min). Then, pellets were washed twice with PBS, dried and snap frozen. Pellets were lysed in 10 mL of modified RIPA lysis buffer (50 mM Tris-HCl pH 7.5, 150 mM NaCl, 1 mM EDTA, 1 mM EGTA, 1% Triton X-100, 0.1% SDS and 1:500 protease inhibitor mixture (Sigma-Aldrich, Saint-Louis, MO, USA), 250U Turbonuclease (Accelagen, San Diego, CA, USA)) at 4 °C for 1 h, and then sonicated (30 s at 35% power, Sonic Dismembrator 500; Fisher Scientific, Nepean, ON, Canada) to disrupt visible aggregates. Lysates were centrifuged at 16,000 rpm (35000× *g*) for 30 min. Clarified supernatants were incubated with 30 µL packed, preequilibrated Streptavidin Sepharose beads (GE) at 4 °C for 3 h. Beads were collected by centrifugation (2000 rpm, 2 min), washed six times with 50 mM ammonium bicarbonate pH 8.3 and treated with TPCK-trypsin (Promega, Madison, WI, USA, 16 h at 37 °C). Supernatants containing the tryptic peptides were collected and lyophilized. Peptides were resuspended in 0.1% formic acid and 1/6 of each sample was analyzed in each MS run.

### 4.4. Flag Affinity Purification

For Flag pull-down experiments, 10 × 150 cm² dishes of FlagBirA*-HRAS, FlagBirA*-NRAS and FlagBirA*-KRAS4B cells were incubated with tetracycline and biotin as above, scraped in PBS, pooled, washed twice in 25 mL PBS and collected by centrifugation at 1000× *g* at 4 °C for 5 min. Cell pellets were stored at −80 °C until lysis. Cell pellets were weighed and 1:4 pellet weight/lysis buffer (by volume) was added. Lysis buffer consisted of 50 mM HEPES-NaOH (pH 8.0), 100 mM KCl, 2 mM EDTA, 0.1% Nonidet P-40, 10% glycerol, 1 mM PMSF, 1 mM DTT and 1:500 protease inhibitor mixture (Sigma-Aldrich, St. Louis, MO, USA). After resuspension, cell pellets were incubated on ice for 10 min, subjected to another freeze–thaw cycle, then centrifugated at 27,000× *g* at 4 °C for 20 min. Supernatants were transferred to fresh 15 mL conical tubes, and 250U Turbonuclease (Accelagen) and 30 µL packed, pre-equilibrated Flag-M2 agarose beads (Sigma-Aldrich) were added. Mixtures were incubated on an end-over-end shaker at 4 °C for 2 h. Beads were pelleted by centrifugation at 1000 rpm (100× *g*) for 1 min and transferred with 1 mL of lysis buffer to a fresh centrifuge tube. Beads were washed once with 1 mL lysis buffer and twice with 1 mL ammonium bicarbonate (NH_4_HCO_3_) rinsing buffer (50 mM NH_4_HCO_3_ pH 8.0, 75 mM KCl). Elution was performed by incubating beads with 150 µL of 125 mM ammonium hydroxide (pH > 11). The elution step was repeated twice more, and the combined eluate was centrifuged at 15,000× *g* for 10 min, transferred to a fresh centrifuge tube and lyophilized. Following overnight trypsin digestion (as above), peptides were resuspended in 0.1% formic acid and 1/6th of each sample was analyzed in each MS run.

### 4.5. Mass Spectrometry

Liquid chromatography (LC) analytical columns (75 m inner diameter) and pre-columns (150 m inner diameter) were made in-house from fused silica capillary tubing from InnovaQuartz (Phoenix, AZ, USA) and packed with 100 Å C18-coated silica particles (Magic, Michrom Bioresources, Auburn, CA, USA). LC-MS/MS was performed using a 120 min reversed-phase buffer gradient running at 250 nL/min (column heated to 40 °C) on a Proxeon EASY-nLC pump iNline with a Q Exactive™ HF Hybrid Quadrupole-Orbitrap™ Mass Spectrometer (Thermo Fisher Scientific). A parent ion scan was performed in the Orbitrap, using a resolving power of 60,000. For protein identification, raw files were converted to the mzXML format using Proteowizard [64], and then analyzed using X!Tandem [65] against Human RefSeq Version 45 (containing 36113 entries). Search parameters were parent MS tolerance of 15 ppm and MS/MS fragment ion tolerance of 0.4 Da, with up to two missed cleavages allowed for trypsin. Methionine oxidation was allowed as variable modification. Data were analyzed using the trans-proteomic pipeline [66] via the ProHits 2.0.0 software suite [67]. Proteins identified with a ProteinProphet cut-off of 0.85 (corresponding to ≤1% FDR) were analyzed with SAINT Express v. 3.3 [68,69]. For BioID, eight control runs were used for comparative purposes and four runs were performed using Flp-In T-REx 293 cells that express FlagBirA*-HRAS, FlagBirA*-NRAS or FlagBirA*-KRAS4B. The eight controls were collapsed to the highest two spectral counts for each hit (Tables S1 and S2).

### 4.6. Interactor Classification

Based on four independent MS runs (two technical replicates and two biological replicates for each bait), bona fide interactors were defined as high confidence protein identifications (ProteinProphet *p* value > 0.85) with a Significance Analysis of INTeractomes (SAINT) score ≥ 0.75. Histones were removed manually. Fold-change was calculated as: log_2_ (1 + spectral count sum Condition A)/(1 + spectral count sum Condition B). The statistical significance of the difference between spectral counts obtained in the different conditions was calculated using the Student’s *t* test. Only hits with log_2_ fold-change ≥ 1 and *p* value ≤ 0.05 were considered as preferential proximal interactor for the corresponding bait.

### 4.7. In Silico Analyses

The PPIs identified were analyzed using the Gene Ontology, Kyoto Encyclopedia of Genes and Genomes (KEGG), Reactome and InterPro bioinformatics tools. The enriched terms with a *p* value ≤ 0.05 were selected, and Revigo [70] was used to summarize highly redundant Gene Ontology terms. The databases BIND, BioGRID and HPRD were used to select the known RAS interacting proteins.

### 4.8. Proximity Ligation Assay (PLA)

FlagBirA*-HRAS, FlagBirA*-NRAS and FlagBirA*-KRAS4B T-REx 293 cells (1.75 × 10^5^) were grown on coverslips in six-well plates for 48 h, and then the pcDNA3 3xHA-tagged RAS substrate candidate was transfected using PolyJet transfection reagent (Signagen, Rockville, MD, USA), according to the manufacturer’s instructions. On Day 1, transfection medium was removed and replaced by fresh medium with tetracycline (1 µg/mL). On Day 2, cells were fixed in ice-cold methanol for 2 min and washed twice in PBS. Then, coverslips were treated according to the manufacturer’s instructions (Duolink In Situ Red starter kit Mouse/Rabbit, Sigma DUO92101). First, samples were saturated using the blocking solution, then mouse anti-Flag (1:500; Euromedex EL-1B11) and rabbit anti-HA (1:3000; Cell signaling C29F4) antibodies were added at 37 °C for 1 h. After two washes in PBS, the PLA minus and plus probes (containing the secondary antibodies conjugated with complementary oligonucleotides) were added at 37 °C for 1 h. The next step allowed the ligation of oligonucleotides only if the two proteins were in close proximity thanks to the ligase during incubation at 37 °C for 30 min. Then, after two washes, the addition of nucleotides and polymerase allowed the amplification by rolling-circle amplification (RCA) reaction using the ligated circle as a template during incubation at 37 °C for 100 min. The amplification solution also contained fluorescently labeled oligonucleotides that hybridized to the RCA product. Then, samples were dried at room temperature in the dark and were mounted with Duolink II Mounting Medium containing DAPI and analyzed using a fluorescence microscope.

### 4.9. Image Acquisition and Analysis

After PLA, coverslips were viewed under a ZEISS Axio Imager 2 microscope. Images were acquired in identical conditions with an 63× objective. For each sample, 100 cells were counted. Analyses and quantifications were performed using the ImageJ software (free access) that allows counting dots on 8-bit images. The plugin “Counter cells” was used to determine cell numbers. Then, the significance of interactions was assessed with the Student’s t-test.

### 4.10. SDS-PAGE and Western Blotting

Cells were lysed by incubation with a commercial cell lysis buffer (CST #9803) on ice for 10 min, followed by centrifugation at 15,000 rpm for 15 min. Supernatants were collected and protein concentration measured with the Micro-Bradford assay (Thermo Scientific). Ten micrograms of proteins were separated on 10% Bis-Tris gels and then transferred to activated PVDF membranes (300 mA for 2 h). Proteins were detected using anti-Flag-M2 (1:5000; Sigma F1804), anti-HA (1:5000; CST #C29F4) and anti-beta-actin (1:5000; Santa Cruz sc-47778) antibodies. Antibody-bound proteins were revealed using an ECL Western blotting substrate (Thermo Scientific) and visualized with a chemiluminescent imaging system.

## 5. Conclusions

In our study, we provide the first large-scale resource and comparison of the wild type RAS paralogs proximal interactors in non-cancerous cells. Altogether, our data show that BioID uniquely provides highly valuable results to decipher RAS biological activities. We found that RAS proximal interactors span multiple cellular compartments and are highly enriched in components involved in cellular energetics and cell cortex. Each paralog was associated with specific features, and we hypothesize that KRAS4B is the most finely regulated paralog, based on the variety of GEFs and GAPs with which it interacts. We believe that our findings will support further studies to better understand how RAS paralogs are differentially involved in discrete cellular processes and, importantly, how RAS mutations in related diseases such as lung cancer or colorectal cancer could impact cell signaling and metabolism. Together, our data could serve as a basis to template oncogenic mechanism investigations and should be mined to design and interpret future RAS-related studies.

## Figures and Tables

**Table 1:** FB-HRAS and FB-NRAS specific functions. The significant FB-HRAS and FB-NRAS-specific functions were determined by Gene Ontology Biological Processes analysis (*p* value < 0.05).

Bait Proteins	Gene Ontology Biological Processes	Count	*p* Value	Identified Proteins
**Specific to FB-HRAS**	Protein transport (GO:0015031)	44	1.47 × 10^−6^	AHCYL1, ANXA2, BSG, BTN3A1, C14orf133, CHMP5-6, DYNLL1, GDI2, GIPC1, NAPA, NDUFAF2, NOP58, PSEN1, RAB11FIP2, RAB12/21/23, SCAMP1, SEC61A1, SLC9A3R1, SNX3/6/12/17, SRC, SRP9, STX3-4/6-7/12, STXBP1, TBC1D10A, TIMM13/23, TMED10, TOMM6, VAMP2/4/8, VPS33A, YKT6, YWHAH
	Single-organism membrane fusion (GO:0060627)	13	4.91 × 10^−6^	ANXA2, STX12, STX3-4/6-7, STXBP1, TC2N, VAMP2/4/8, VAT1, YKT6
	Regulation of vesicle-mediated transport (GO:0060627)	19	2.40 × 10^−5^	ANXA2, B2M, CHMP6, CNN2, NAPA, NCS1, RAB21, RDX, SNX3/6/12/17, SRC, STX4, TBC1D10A, TC2N, VAMP2/8
	Endosomal transport (GO:0016197)	14	6.98 × 10^−5^	C14orf133, CHMP5-6, RAB12/21, RDX, SNX3/6/12/17, STX6, TBC1D10A, VPS33A, YKT6
	Exocytosis (GO:0006887)	17	1.00 × 10^−4^	CHMP6, NAPA, NCS1, PSEN1, RAB11FIP2, SCAMP1, SNX6, STX3-4, STXBP1, TC2N, TMED10, VAMP2/4/8, VPS33A, YKT6
**Specific to FB-NRAS**	Transmembrane transport (GO:0055085)	15	1.41 × 10^−3^	AKT2, ANK3, CNKSR3, EBP, SLC2A1, SLC5A6, SLC16A10, SLC25A1, SLC30A5-6, SLC35A2/E1, UBB, ZDHHC13/17

FB, FlagBirA* dual tag.

**Table 2:** FB-HRAS and FB-KRAS specific protein domains. The significant FB-HRAS and FB-KRAS-specific protein domains were determined by InterPro analysis (*p* value < 0.05).

Bait Proteins	InterPro Protein Domains	Count	*p* Value	Identified Proteins
**Specific to FB-HRAS**	Syntaxin/epimorphin, conserved site (IPR006012)	5	2.07 × 10^−3^	STX3-4/6-7/12
	t-SNARE (IPR010989)	5	2.16 × 10^−3^	STX3-4/6-7/12
	Synaptobrevin (IPR001388)	4	8.66 × 10^-3^	VAMP2/4/8, YKT6
**Specific to FB-KRAS**	CRAL-TRIO domain (IPR001251)	4	2.10 × 10^−2^	NF1, MOSPD2, KALRN, TRIO

FB, FlagBirA* dual tag.

## Supplementary Materials

The following are available online at https://www.mdpi.com/2072-6694/12/11/3326/s1, Figure S1: Comparison of the RAS networks identified by BioID in this study and by Kovalski et al. (2019) [39]. Figure S2: RAS interactors identified by BioID and involved in known RAS signaling cascades. Figure S3: Identification of preferential interactors for each RAS paralog. Figure S4: Validation by PLA of RAS paralog-specific interactions. Figure S5: Uncropped Western blot in Figure 6A showing FB-RAS expression. Figure S6: Uncropped Western blot in Figure 6A showing HA-BRAF expression. Figure S7: Uncropped Western blot in Figure S4A showing HA-RAP2A expression. Figure S8: Uncropped Western blot in Figure S4A showing HA-SHKBP1 expression. Figure S9: Uncropped Western blot in Figure S4A showing HA-PGD expression. Figure S10: Uncropped Western blot in Figure S4B showing HA-S100A11 expression. Figure S11: Uncropped Western blot in Figure S4B showing HA-EBP expression. Table S1: FB-HRAS, FB-NRAS and FB-KRAS BioID results. Table S2: FB-HRAS, FB-NRAS and FB-KRAS IP-MS results. Table S3: Gene Ontology analysis of the proteins specifically detected with BioID or IP-MS. Table S4: Top ten enriched Gene Ontology Biological Process for the cytosolic proteins specifically detected with BioID or IP-MS. Table S5: KEGG analysis. Table S6: Total spectral count log_2_ ratio for each pairs of RAS paralogs. Table S7: Identification of the highly preferential proximal interactors of FB-KRAS, FB-NRAS or FB-HRAS. Table S8: Selected interactors for proximity ligation assay. Table S9: Primers, destination plasmids, cloning sites and DNA templates.
