# Supplementary material for: Proximal Protein Interaction Landscape of RAS Paralogs
Source: Cancers (Basel). 2020 Nov 11;12(11):3326. doi: 10.3390/cancers12113326 (PMC7696408; doi:10.3390/cancers12113326)
Supplement: Supplementary file 1 [file cancers-12-03326-s001.zip › Cancers - B¿aganton et al - Supplementary Materials (figures).pdf]

cancers

MDPI

Supplementary Materials

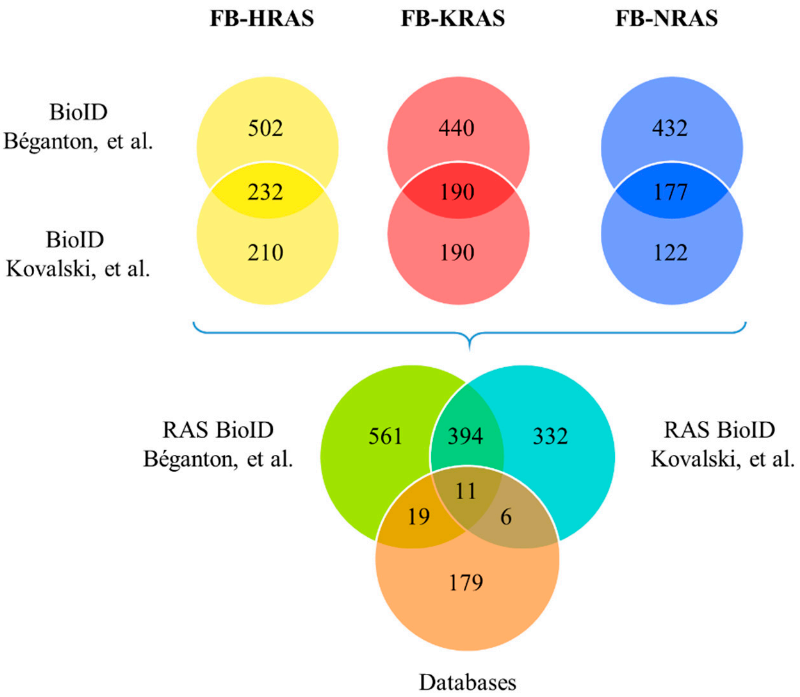

Figure S1. Comparison of the RAS networks identified by BioID in this study and by Kovalski et al. (2019) [39]. The interacting partners in the two BioID datasets were compared for each RAS paralog, and then collapsed and compared to the RAS partners reported in the BIND, BioGRID and HPRD databases. FB, FlagBirA* dual tag.

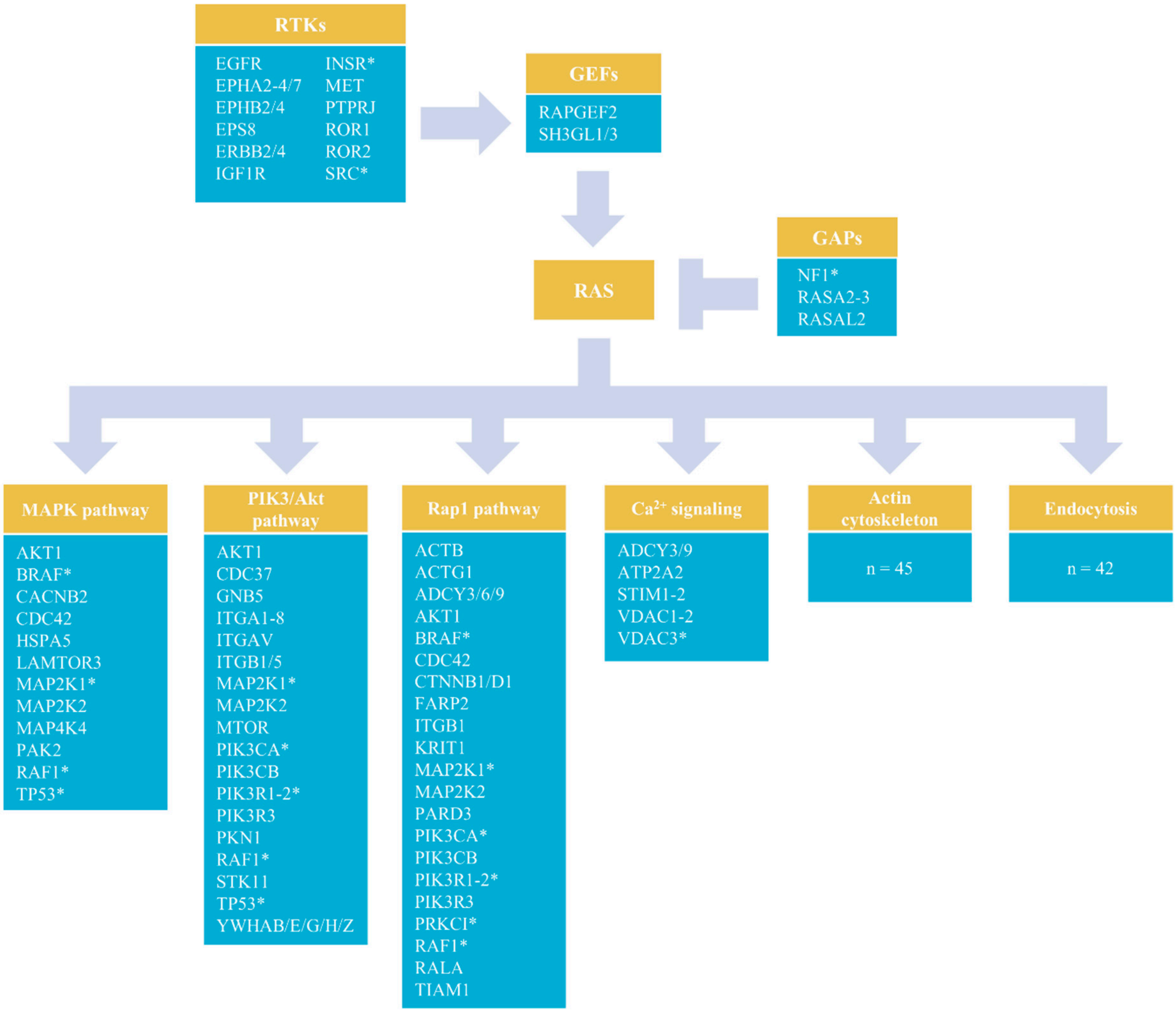

Figure S2. RAS interactors identified by BioID and involved in known RAS signaling cascades. The listed proteins are mapped according to their protein family (involved in RAS signaling) or to their implication in RAS cellular pathways. The protein families involved in RAS signaling are: receptor tyrosine kinases (RTKs), guanine nucleotide exchange factors (GEFs), and GTPase-activating proteins (GAPs). The RTKs, GEFs and GAPs are compiled from literature data. The KEGG annotation was used to identify proteins from the mitogen-activated protein kinase (MAPK) pathway (hsa04010), PIK3/Akt pathway (hsa04151), Rap1 pathway (hsa04015), calcium signaling pathway (hsa04020), regulation of actin cytoskeleton pathway (hsa04810), and endocytosis pathway (hsa04144). Proteins annotated with an asterisk (*) were previously identified as RAS physical interactors (BIND, BioGRID, and HPRD databases).

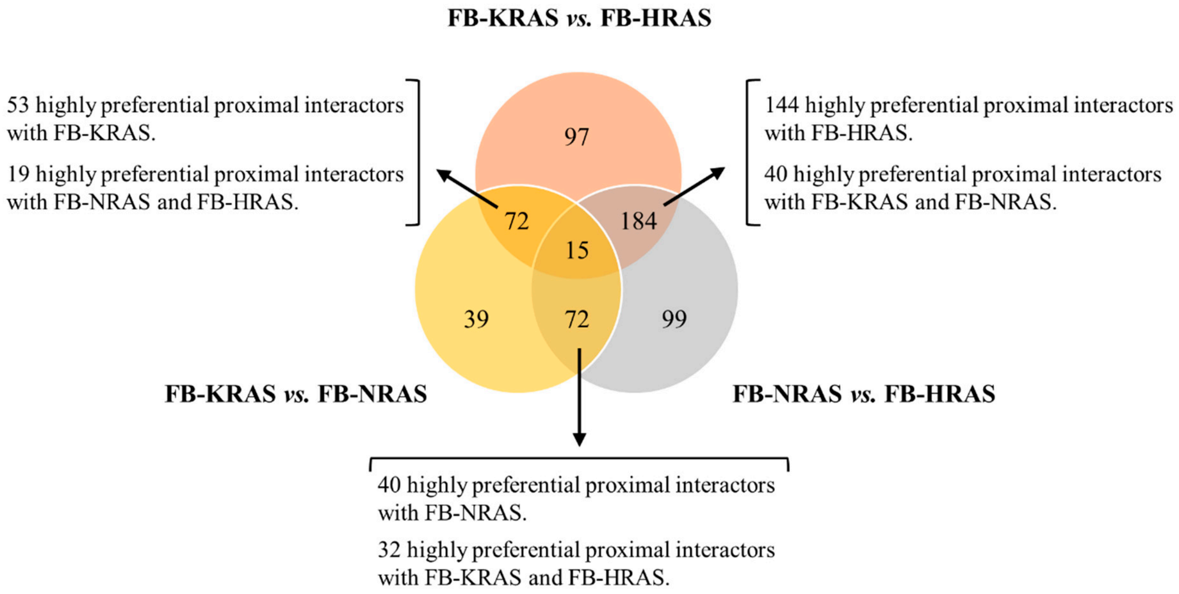

Figure S3. Identification of preferential interactors for each RAS paralog. The comparison of the identified interactors between the indicated baits allowed clustering the highly preferential interactors for each paralog. FB, FlagBirA* dual tag.

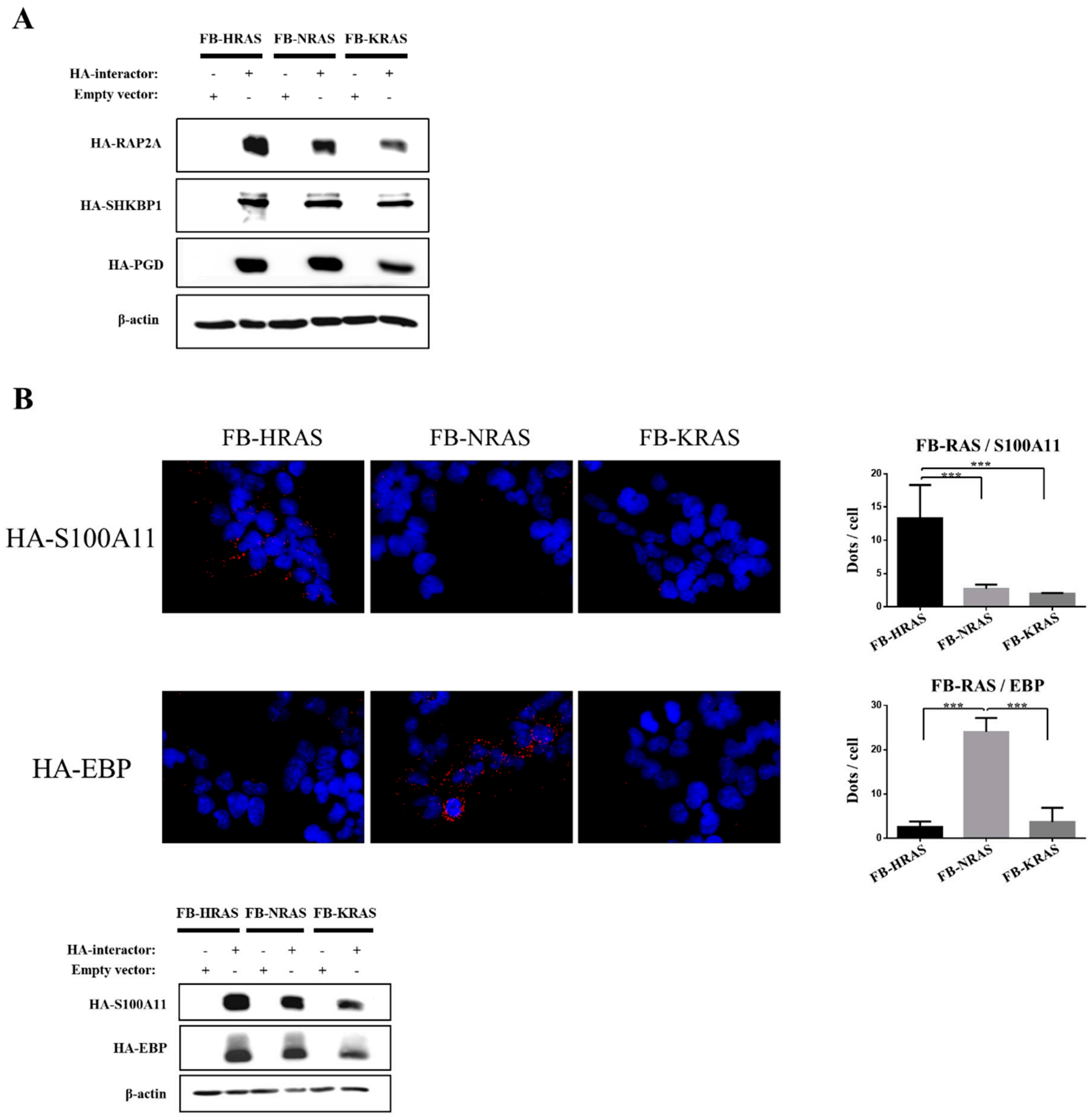

Figure S4. Validation by PLA of RAS paralog-specific interactions. **(A)** Validation of the ectopic expression of HA-RAP2A, -SHKBP1 and –PGD; **(B)** PLA detection of the specific interaction of FB-HRAS and FB-NRAS with HA-tagged S100A11 and EBP, respectively. PLA was performed with anti-Flag and -HA antibodies. The detected complexes are represented by red dots and nuclei were counterstained with DAPI (blue). Quantification of the dots per cell was performed by using Image J and the plugin ''Counter cells''. Data are the mean±SEM 100 cells/condition. The expression of the HA-tagged proteins was validated by western blotting. ***, p value ≤ 0.0001 (Student's t-test); FB, FlagBirA* dual tag.

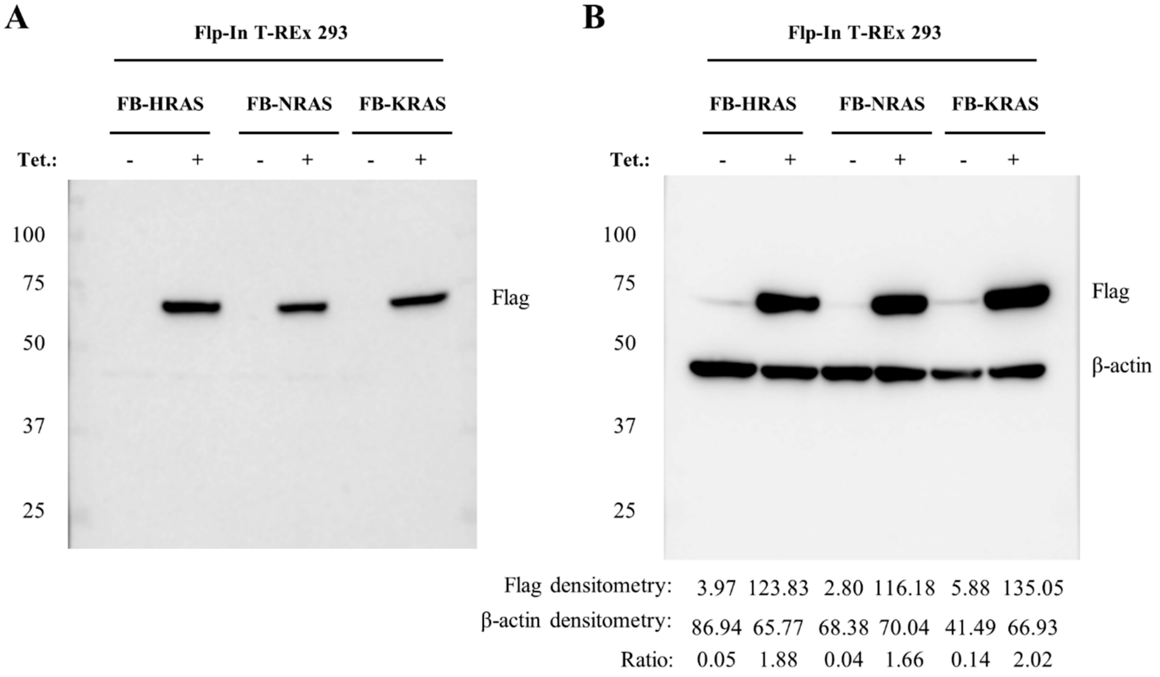

Figure S5. Uncropped Western blot in Figure 6A showing FB-RAS expression. Images are the merge of the last signal acquisition and bright field in order to show the protein ladder. Signals in the dynamic range were used to calculate the densitometries and ratios. **(A)** Expression of the FB-RAS proteins; **(B)** Detection of β-actin as loading control. FB, FlagBirA* dual tag; Tet.,tetracycline.

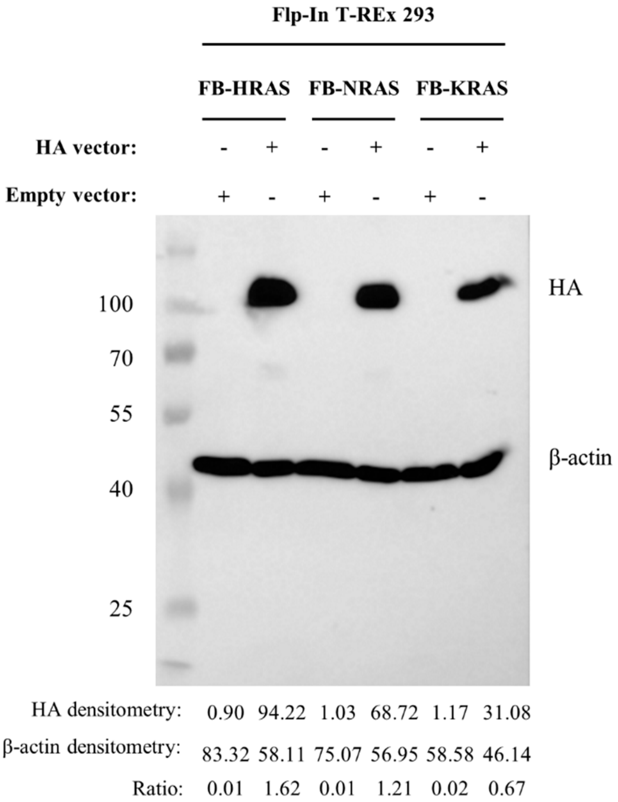

Figure S6. Uncropped Western blot in Figure 6A showing HA-BRAF expression. β-actin was detected as loading control. The image is the merge of the last signal acquisition and bright field in order to show the protein ladder. Signals in the dynamic range were used to calculate the densitometries and ratios. FB, FlagBirA* dual tag.

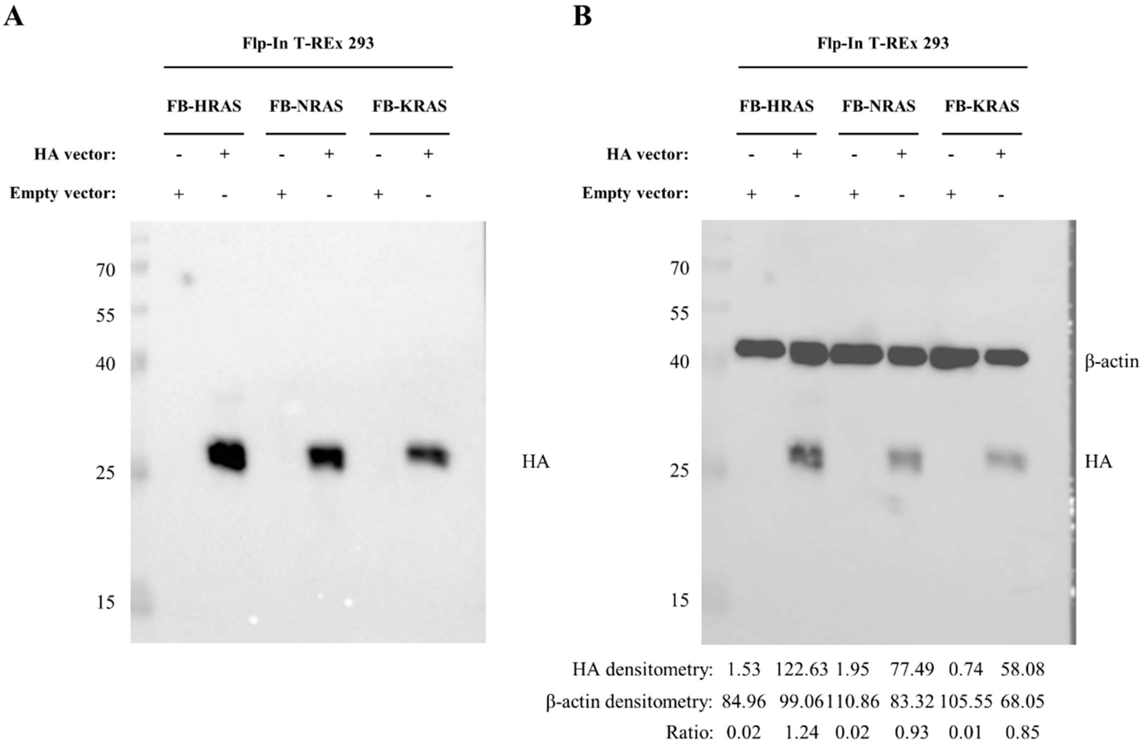

Figure S7. Uncropped Western blot in Figure S4A showing HA-RAP2A expression. Images are the merge of the last signal acquisition and bright field in order to show the protein ladder. Signals in the dynamic range were used to calculate the densitometries and ratios. **(A)** Expression of HA-RAP2A; **(B)** Detection of β-actin as loading control. FB, FlagBirA* dual tag.

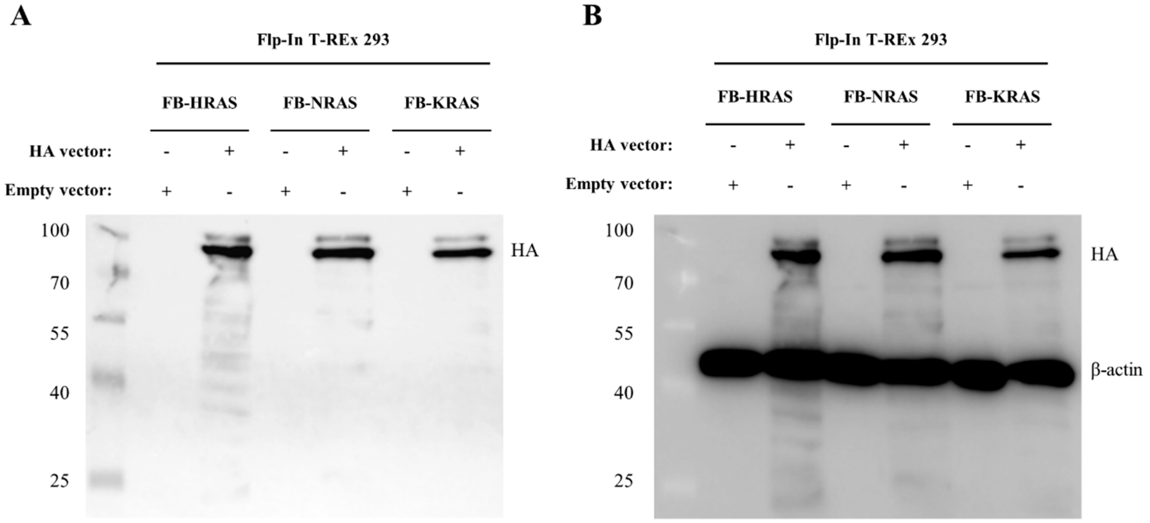

HA densitometry:	1.33	65.99	2.62	76.33	2.61	49.68
β-actin densitometry:	97.48	101.64	101.65	94.29	88.87	81.81
Ratio:	0.01	0.65	0.03	0.81	0.03	0.61

Figure S8. Uncropped Western blot in Figure S4A showing HA-SHKBP1 expression. Images are the merge of the last signal acquisition and bright field in order to show the protein ladder. Signals in the dynamic range were used to calculate the densitometries and ratios. **(A)** Expression of HA-SHKBP1; **(B)** Detection of β-actin as loading control. FB, FlagBirA* dual tag.

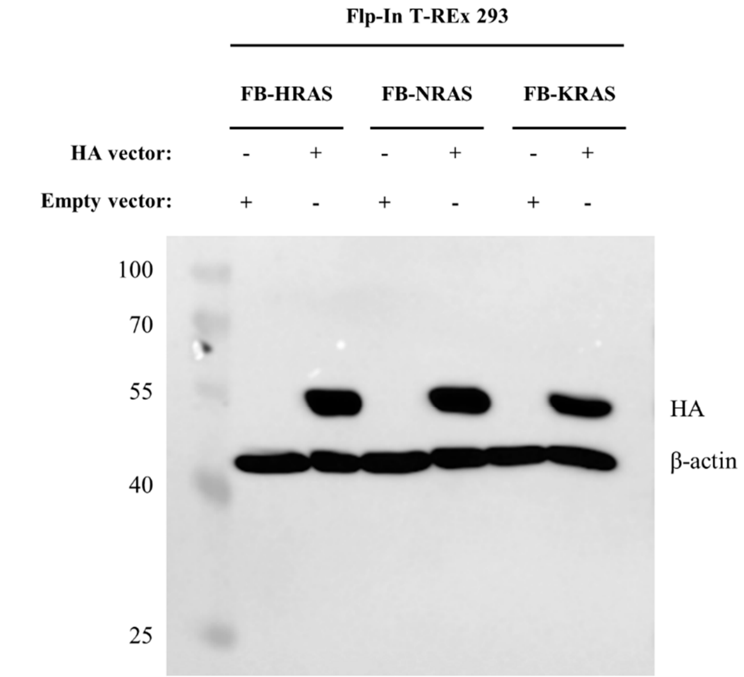

HA densitometry:	0.24	66.36	0.53	74.63	1.71	37.78
β-actin densitometry:	83.15	67.83	93.93	85.47	88.05	86.98
Ratio:	0.00	0.98	0.01	0.87	0.02	0.43

Figure S9. Uncropped Western blot in Figure S4A showing HA-PGD expression. β-actin was detected as loading control. The image is the merge of the last signal acquisition and bright field in order to show the protein ladder. Signals in the dynamic range were used to calculate the densitometries and ratios. FB, FlagBirA* dual tag.

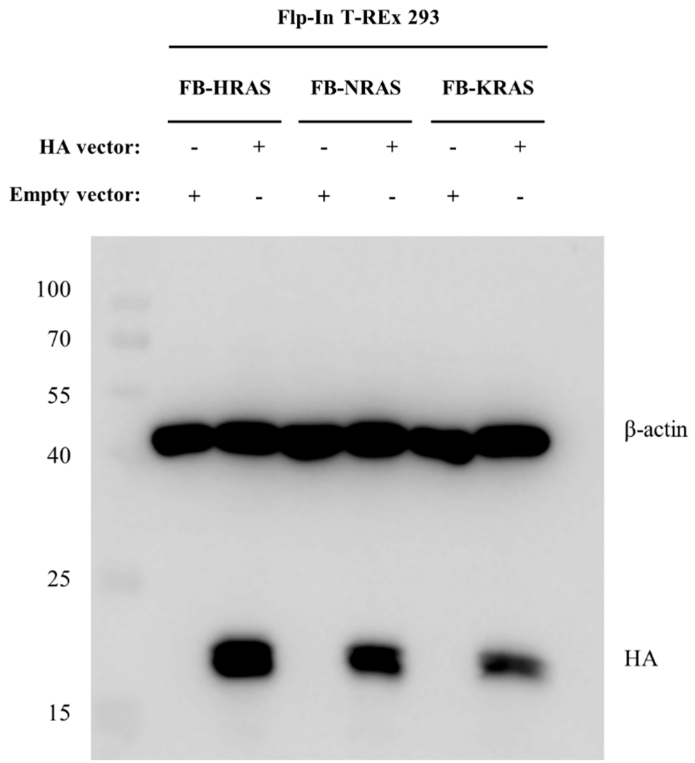

HA densitometry:	2.08	82.06	1.44	43.88	0.69	26.86
β-actin densitometry:	81.43	98.32	101.40	100.89	91.80	103.83
Ratio:	0.03	0.83	0.01	0.43	0.01	0.26

Figure S10. Uncropped Western blot in Figure S4B showing HA-S100A11 expression. β-actin was detected as loading control. The image is the merge of the last signal acquisition and bright field in order to show the protein ladder. Signals in the dynamic range were used to calculate the densitometries and ratios. FB, FlagBirA* dual tag.

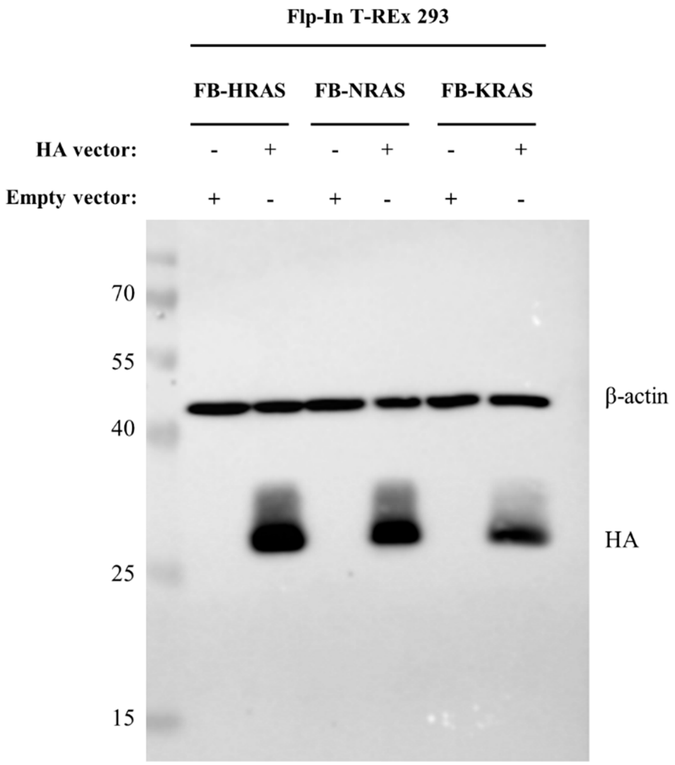

HA densitometry:	1.20	164.58	5.14	131.78	3.07	87.55
β-actin densitometry:	95.55	82.16	85.67	59.28	76.84	79.82
Ratio:	0.01	2.00	0.06	2.22	0.04	1.10

Figure S11. Uncropped Western blot in Figure S4B showing HA-EBP expression. β-actin was detected as loading control. The image is the merge of the last signal acquisition and bright field in order to show the protein ladder. Signals in the dynamic range were used to calculate the densitometries and ratios. FB, FlagBirA* dual tag.
